# Cost effectiveness and health-related quality of life of chemoradiotherapy versus radiation therapy alone in elderly head and neck cancer patients

**DOI:** 10.1007/s00066-022-01975-6

**Published:** 2022-07-14

**Authors:** Tanja Sprave, Vivek Verma, Alexander Fabian, Alexander Rühle, Dimos Baltas, Anca-Ligia Grosu, Nils H. Nicolay

**Affiliations:** 1 Department of Radiation Oncology, University of Freiburg – Medical Center, Freiburg, Germany; 2 German Cancer Consortium (DKTK) Partner Site Freiburg, German Cancer Research Centre (DKFZ), Heidelberg, Germany; 3 Department of Radiation Oncology, University of Texas M.D. Anderson Cancer Center, Houston, TX USA; 4 Department of Radiation Oncology, University Hospital Kiel, Kiel, Germany

**Keywords:** Head and neck squamous cell carcinoma, Geriatric patients, Chemoradiation, EQ‑5D, Quality-adjusted life year

## Abstract

**Purpose:**

Radiotherapy (RT) constitutes a mainstay in the treatment of elderly patients with head and neck cancer (HNC), but use of simultaneous chemoradiotherapy (CRT) remains controversial. We have conducted a prospective analysis based on real-world patient data to examine the health-related quality of life (HRQoL) and cost effectiveness (CE) of CRT vs. RT in elderly HNC patients.

**Methods:**

Eligible participants ≥ 65 years treated in a large tertiary cancer center between July 2019 and February 2020 who completed the validated EQ-5D-5L questionnaire (health state index [HI] and visual analog scale [VAS]) before and after RT were included. CE referred to direct medical costs, including diagnosis-related group (DRG)-based billings for inpatients and uniform assessment standard (EBM)-based costs for outpatients. The primary endpoint was cost (euros [€]) per quality-adjusted life year (QALY). The incremental cost-effectiveness ratios (ICERs) were calculated. Costs and QALYs were not discounted for short overall survival (OS).

**Results:**

Baseline HRQoL was 0.878 (±0.11) in the CRT group and 0.857 (±0.17) in the RT group. Upon completion of therapy, HRQoL amounted to 0.849 (±0.14) in the CRT and 0.850 (±0.13) in the RT group. The mean treatment-related cost in the CRT cohort was €22,180.17 (±8325.26) vs. €18,027.87 (±26,022.48) in the RT group. The corresponding QALYs amounted to 2.62 in the CRT and 1.91 in the RT groups. The ICER was €5848.31.

**Conclusion:**

This is the first analysis from the German health care system demonstrating that the addition of chemotherapy to RT for selected elderly HNC patients is cost effective and not associated with a significant HRQoL decline.

## Introduction

The incidence of head and neck cancers (HNC) in elderly individuals is expected to rise considerably over the next decades [1, 2]. Curative treatment of elderly HNC patients often requires multimodal approaches including surgery, radiotherapy (RT), or concomitant chemoradiation (CRT), but therapy is often complicated by reduced patient performance or comorbidities. Additionally, treatment-related toxicities commonly require intensified medical procedures and additional supportive care in elderly patients, resulting in substantial healthcare resource requirements and costs [3–6]. In this respect, it is important to compare increased treatment-related costs to benefits in treatment outcomes and patients’ health-related quality of life (HRQoL) [7–9]. A key controversy for elderly patients undergoing definitive RT as a curative treatment relates to the addition of concomitant chemotherapy: although concomitant systemic treatment results in an additional improvement in patient survival, this benefit decreases with age and was no longer detectable in patients ≥ 70 years in the MACH-NC meta-analysis [10, 11]. Based on these data, it has long been debated whether age alone is a determining factor for treatment choice in elderly HNC patients [12, 13]. Considering the onset of potentially severe chemotherapy-induced toxicities in this vulnerable patient group, the questionable benefits of adding chemotherapy must be carefully weighed against its negative impact on patients’ quality of life and resulting cost effectiveness (CE) [14, 15]. To date, only very limited data are available investigating HRQoL and resulting CE in elderly HNC patients [16–18]; for example, the French ELAN initiative is currently investigating the benefit of different chemotherapy protocols for fit and unfit elderly HNC patients with recurrent or metastatic disease within their trial program [19, 20].

Currently, a number of validated and internationally recognized approaches exist for measuring HRQoL based on the calculation of quality-adjusted life years (QALYs) [21–25]. Combining gains in patient outcomes and quality of life in a single metric allows comparison between different interventions as well as computation of incremental costs and CE [26, 27]. In this context, the EQ-5D, as a standardized measure of HRQoL, has been widely employed for health economy evaluations in the elderly [28, 29].

Using this validated tool, we aimed to prospectively evaluate and compare HRQoL and resulting CE of curative platinum-based CRT compared to RT alone in elderly HNC patients.

## Patients and methods

### Patients and treatment

The current study was approved by the Independent Ethics Committee of the Medical Faculty at the University of Freiburg (record no. 389/19), and written informed consent was obtained from all patients who agreed to participate. All patients aged ≥ 65 years receiving curative RT for HNC between July 2019 and February 2020 at the Department of Radiation Oncology, University Medical Center Freiburg, were screened for this analysis.

Demographic and treatment characteristics were obtained from the electronic patient records. Based on imaging and pathology, tumor nomenclature was performed according to the 8th edition of the TNM classification of malignant tumors. Individuals with a smoking history of at least 10 pack years were considered as smokers.

Treatment for all elderly HNC patients was based on multidisciplinary tumor board recommendations. Briefly, curative CRT was recommended in the definitive or adjuvant setting for locally advanced and inoperable tumors. Adjuvant cases were eligible for CRT based on histologically confirmed positive resection margins and/or extranodal/extracapsular spread. The standard dose for definitive CRT was 70 Gy EQD_2_ to the primary tumor, whereas patients undergoing adjuvant or repeat RT received 60–66 Gy EQD_2_ to the tumor cavity. RT was performed using intensity-modulated radiotherapy (IMRT) and image guidance (IGRT).

### Health-related quality of life questionnaires

All patients aged ≥ 65 years receiving curative RT for HNC between July 2019 and February 2020 at the Department of Radiation Oncology, University Medical Center Freiburg, were asked to complete the EQ-5D-5L questionnaire before and after RT. The EQ-5D-5L is a generic quantitative measure for the generated health state index (HI) score and perceived health. It has been validated and is recommended for health technology assessment by the National Institute for Health and Clinical Excellence. The first descriptive component of the questionnaire pertains to the health state index (HI), as related to the five dimensions of mobility, self-care, daily activities, pain, and anxiety. Each parameter can be quantified on a five-grade scale (no, slight, moderate, severe, and extreme problems/inability). The second component of the questionnaire provides a visual analogue scale (VAS) for perceived overall health ranging from 0 to 100 (corresponding to the worst to the best imaginable health).

### Statistical analysis

Statistical analyses of clinical and sociodemographic data were conducted with IBM SPSS Statistics software version 25 (IBM, Armonk, NY, USA). Descriptive statistics of the HI and VAS values were done in accordance with published guidelines. Normal distributions were tested using the Shapiro–Wilk test. Mann–Whitney U tests and chi-square tests were performed to assess potential differences between the different treatment groups and within each group. A two-tailed *p* < 0.05 was considered statistically significant for all analyses.

CE analysis focused on direct costs from the hospital’s perspective, as all patients in our cohort were retired and did not incur significant external costs. The detailed radiotherapy-associated treatment costs for each patient were based on diagnosis-related groups (DRG) of the German statutory health insurance for all inpatient treatments and based on the uniform assessment standard (*einheitlicher Bewertungsmaßstab* [EBM]) for 2019 and 2020 for all outpatient treatments. Incremental cost-effectiveness ratios (ICERs) were calculated according to the following formula:

$$(\text{costs}_{\mathrm{CRT}}-\text{costs}_{RT})/(\mathrm{QALY}_{\mathrm{CRT}}-\mathrm{QALY}_{RT})$$

## Results

### Patient population

**Fig. 1:**
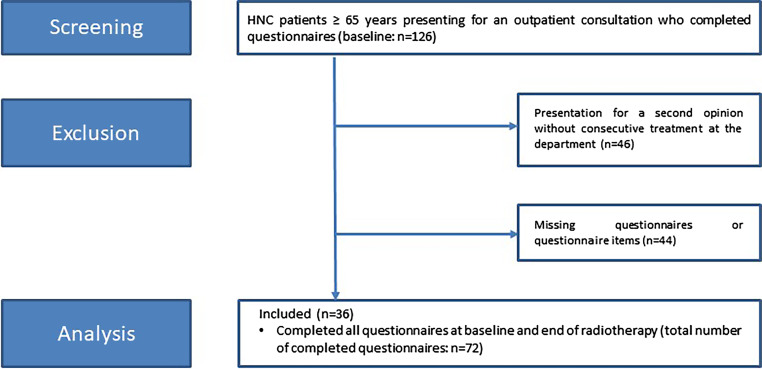
Flow chart of screening and inclusion procedures for this analysis. *HNC* head and neck cancer

A total of 126 HNC patients ≥ 65 years scheduled for concomitant CRT presented to our center between July 2019 and February 2020 and were screened for this analysis. 46 patients were excluded as they presented for a second opinion and did not undergo RT at our department. 44 additional patients did not fill out all items in the baseline and posttreatment questionnaires and were excluded from this study. For 36 patients, all items in both questionnaires were available for further analysis (Fig. 1).

Based on tumor stage and/or histopathological risk factors such as incomplete resection or extracapsular spread, CRT had been recommended in all patients, but due to pre-existing comorbidities, concomitant chemotherapy was omitted in 18 patients (50%) as per the discretion of the treating physicians. Patients were predominantly male in both groups (CRT: *n* = 13, 72.2%; RT: *n* = 12, 66.7%; *p* = 0.748) with a median Charlson Comorbidity Index of 7 points in each group (*p* = 0.714). The median age was significantly lower in the CRT group and amounted to 71 years (65–88 years) vs. 81 years in the RT group (range 65–89 years, *p* = 0.047; Table 1). Most patients were smokers (CRT: *n* = 12, 66.7%; RT: *n* = 7, 38.9%; *p* = 0.095; chi-square test).

**Table 1:** Baseline characteristics of the study population

	RT	RCT	
	*n* (%)	*n* (%)	*p*-value
*All (n* *=* *36)*	18 (50)	18 (50)	
*Age (years; median, range)*	81 (65–89)	71 (65–88)	0.047^a^
*Sex*			
Female	6 (33.3)	5 (27.8)	0.748^b^
Male	12 (66.7)	13 (72.2)	
*Smoking*			
No	11 (61.1)	6 (33.3)	0.095^b^
Yes	7 (38.9)	12 (66.7)	
*Localization*			
Nasopharynx	1 (5.6)	2 (11.1)	0.642^b^
Oropharynx	3 (16.7)	7 (38.9)	
Hypopharynx	5 (27.8)	4 (22.2)	
Oral cavity	1 (5.6)	1 (5.6)	
Salivary glands	2 (11.1)	1 (5.6)	
Others	6 (33.3)	3 (16.7)	
*Histology*			
Squamous cell carcinoma	16 (88.8)	16 (88.8)	0.388^b^
Adenocarcinoma	1 (5.6)	0	
Undifferentiated	0	1 (5.6)	
Others	1 (5.6)	1 (5.6)	
*Grading*			
1	0	0	0.026^b^
2	17 (94.4)	10 (55.6)	
3	1 (5.6%)	7 (38.9)	
4	0	1 (5.6)	
*Extranodal/extracapsular spread*			
No	13 (72.2)	17 (94.4)	0.074^b^
Yes	5 (27.8)	1 (5.6)	
*Radiotherapy*			
Definitive	11 (61.1)	16 (88.9)	0.054^b^
Adjuvant	7 (38.9)	2 (11.1)	
Re-irradiation	4 (22.2)	4 (22.2)	
*Radiotherapy technique*			
IMRT	18 (100)	18 (100)	
*Concomitant chemotherapy*			
Yes	0	18 (100)	
*Inpatient days (median, range)*	0 (0–50)	26 (9–55)	0.002^a^
Yes	10 (55.6)	18 (100)	
No	8 (44.4)	0	
*Secondary malignancy*			
Yes	5 (27.8)	8 (44.4)	0.298^b^
No	13 (72.2)	10 (55.6)	
*Charlson score (median, range) at baseline*	7 (5–12)	7 (4–17)	0.714^a^

^a^Mann–Whitney U test

^b^Pearson chi-square test

In the CRT group, 16 patients (88.9%) received definitive and 2 patients (11.1%) received adjuvant treatment. In the RT group, 11 (61.1%) and 7 patients (38.9%) received definitive and adjuvant therapy, respectively (*p* = 0.054; chi-square test). All patients in the CRT group required an inpatient admission for median 26 (range 9–55) days, whereas in the RT group, only 10 patients (55.6%) required supportive inpatient admission (*p* = 0.002).

### Acute toxicities

Acute RT-related toxicities were assessed at the end of treatment according to the National Cancer Institute’s Common Terminology Criteria for Adverse Events (CTCAE v5.0). In summary, rates of treatment-related higher-grade (≥ grade 3) acute toxicities were similar, and included dysphagia in 2 patients in each group, along with oral mucositis and xerostomia in 1 patient per group. There were no acute grade 4 or 5 toxicities in either group. Detailed toxicity rates are shown in Table 2.

**Table 2:** Acute radiotherapy-related toxicities according to the Common Terminology Criteria for Adverse Events (CTCAE v5.0)

All (*n* = 36)	RT (*n* = 18)				RCT (*n* = 18)				
CTCAE grade	0	1	2	3	0	1	2	3	*p*-value
Dermatitis	0	10	8	0	1	13	4	0	0.256
Dysphagia	0	10	6	2	2	9	5	2	0.543
Dysgeusia	4	10	4	0	3	9	5	0	0.761
Nausea	10	0	1	0	14	3	1	0	0.193
Mucositis	0	12	5	1	2	8	7	1	0.372
Xerostomia	3	13	1	1	2	13	2	1	0.912
Pain	8	5	5	0	10	4	3	0	0.669
Hoarseness	16	2	0	0	15	3	0	0	0.630

### Health state index and visual analog scale

For the CRT cohort, the mean EQ-5D-5L HI score before and after treatment was 0.878 ± 0.11 and 0.849 ± 0.14, respectively. In the group with RT alone, the HI scores amounted to 0.857 ± 0.17 and 0.850 ± 0.13, respectively (Table 3). The corresponding mean VAS scores were 67.61 ± 21.4 and 65.0 ± 22.9 in the CRT group vs. 72.50 ± 24.5 and 62.78 ± 16.2 in the RT group, respectively (Table 3). Changes in the HI were not significant in either group (*p* = 0.350 for the CRT cohort and *p* = 0.370 for the RT cohort). Similarly, HI did not significantly differ between groups at baseline (*p* = 0.999) or at completion of therapy (*p* = 0.844).

**Table 3:** EQ-5D-5L health index and VAS values for elderly CRT and RT patients

Baseline			RT end		
	Mean (SD)	*p*-value between groups	Mean (SD)	*p*-value between groups	*p*-value within group
*HI*					
RT (*n* = 18)	0.857 (0.17)	0.999^ac^	0.850 (0.13)	0.844^ad^	0.370^b^
RCT (*n* = 18)	0.878 (0.11)		0.849 (0.14)		0.350^b^
*VAS*					
RT (*n* = 18)	72.50 (24.5)	0.372^ac^	62.78 (16.2)	0.389^ad^	0.015^b^
RCT (*n* = 18)	67.61 (21.4)		65.0 (22.9)		0.361^b^

*RT* radiotherapy, *RCT* chemoradiotherapy

^a^Mann–Whitney U test

^b^Wilcoxon signed-rank test

^c^RT vs. RCT group at baseline

^d^RT vs. RCT group at the end of therapy

As shown in Table 3, there was no significant decline in the VAS scores within the CRT group from baseline to the end of treatment (*p* = 0.361); however, VAS scores in the RT group significantly deteriorated from baseline to completion (*p* = 0.015). The differences in VAS score between the CRT and the RT groups were not significant at either baseline (*p* = 0.372) or therapy completion (*p* = 0.389).

The impact of clinical and pathological factors (chemotherapy, inpatient stay, extracapsular spread, smoking status, comorbidity index, total radiation dose, and acute treatment-related toxicities) on the treatment-related change in HRQoL was examined using multiple regression analysis. Increasing duration of inpatient stay decreased (*p* = 0.002) and chemotherapy (*p* = 0.041) increased intervals of HRQoL.

### Direct costs

The mean direct treatment costs in the CRT group amounted to 22,180.17 ± 8,325.26 € vs. 18,027.87 ± 26,022.48 € in the RT group. Treatment costs are outlined in Table 4.

**Table 4:** Real-world costs and QALYs for each group

	RT	CRT	
All (*n* = 36)	Mean (SD)	Mean (SD)	*p*-value
Health index	0.850 (0.13)	0.849 (0.14)	0.844^a^
Costs (€)	18,027.87 (26,022.48)	22,180.17 (8325.26)	–
Overall survival (months)	27	37	–
QALY’s	0.850 × 27 = 22.95 : 12 = **1.91**	0.849 × 37 = 31.41 : 12 = **2.62**	–

The bold values are the actual QALYs. The other numbers only serve as calculation values.

^a^Mann–Whitney U test

### Quality-adjusted life years and incremental cost-effectiveness ratios

Overall survival (OS) estimation for both groups was based on published in-house data of elderly patients undergoing RT or CRT [30]. In this study, median OS in the CRT group was 37 months vs. 27 months in the RT group. The median OS in the CRT group at 37 months corresponded to 2.62 QALYs, whereas the OS in the RT group of 27 months corresponded to 1.91 QALYs. ICERs calculated as the incremental costs required for an additional QALY were €5,848.31, suggesting strong cost effectiveness for adding chemotherapy to RT in elderly HNC patients (Table 4). Costs and QALYs were not discounted for short OS [31].

## Discussion

Using prospective real-world data from the German healthcare system, this study demonstrated for the first time that curative CRT is highly cost effective compared to RT for selected elderly HNC patients [32]. To the best of our knowledge, no evidence has been published to date regarding the CE of cisplatin-based concomitant CRT for elderly HNC patients; therefore, no comparisons are possible regarding the CE in other healthcare systems. Considering the previously reported relevance of age regarding the benefits of concomitant chemotherapy in elderly HNC patients, our data support the notion that additional factors such as patient performance and comorbidities may strongly influence oncological outcomes and hence CE [10, 33]. One previous analysis comparing cisplatin-based CRT and RT from Brazil that did not focus on elderly HNC patients demonstrated the CE with an ICER of $3303 per life-year gained [34]. These findings are comparable to our results in elderly HNC patients, with an ICER of €5848.

We believe that our findings may have an impact on decision-making processes regarding the choice of therapy in elderly HNC patients. It has to be considered that in our cohort, HRQoL did not significantly deteriorate towards the end of therapy despite increasing treatment-associated toxicities (Table 3). Additionally, highly comparable HI scores were calculated for patients undergoing CRT or RT upon completion of therapy. HI scores were in line with previously published data on HNC patients from our group, demonstrating HI scores of 0.84 directly after treatment and 0.85 at the 3‑ and 6‑month follow-up examinations [35]. Notably, average HI values in the general German elderly population (≥ 65 years) range between 0.80 and 0.85 depending on the age cohort, and are strongly comparable to the HI data obtained in this study [36–38]. Calculation of HI values may depend on the type of HRQoL survey, but it has been demonstrated in a large Canadian analysis that converting HRQoL scores obtained from EORTC QLQ C30 and H&N35 questionnaires into EQ-5D-based scores results in highly comparable HI values in HNC patients [39, 40]. Similarly, the published VAS score for the general German elderly population amounts to 73.2, and gender-specific VAS values in females and males ≥ 70 years have been reported at 71.7 and 70.8, respectively, demonstrating data comparable to the patients included in this analysis [36, 41]. Comparable HRQoL values between the general German population and surviving elderly HNC patients undergoing CRT or RT have also been reported previously [15].

As the EQ-5D-5L questionnaires are limited to testing only few health domains, there is a risk for demonstrating a premature optimal health level. Therefore, despite the ease of use of EQ-5D, the questionnaire has been criticized for its ceiling effect, and modifications have been performed to reduce this risk [42–46]. Compared to the previous EQ-5D-3L, the current EQ-5D-5L questionnaires demonstrated superior assessments and have therefore been recommended for general use as well as for HRQoL measurements in vulnerable cohorts with cancer and multiple comorbidities [45, 47]. The exact comparison of individual QoL questionnaires remains to be elucidated in a wider real-world clinical setting [48]. Especially considering the specific characteristics of elderly cancer patients, the performance of the EQ-5D questionnaire needs to be further evaluated against other QoL tools [36, 49].

A standardized recommendation for interpretation of patient-reported treatment-induced changes to evaluate the efficacy of an intervention in oncological patients has been established [50]. Notably, for elderly HNC patients, the lack of significant changes over time may represent a treatment benefit, as deteriorations resulting from locoregional tumor progression can be excluded [35, 50]. Thus, differences in HI values between baseline and the completion of treatment were calculated, and additional clinical and pathological factors influencing these treatment-induced changes were taken into consideration. Based on these analyses, we could demonstrate that an increasing duration of inpatient treatment reduced, and the application of concomitant chemotherapy increased intervals, suggesting a beneficial effect of prolonged inpatient stays and an adverse influence of concomitant chemotherapy administration on elderly HNC patients’ HRQoL. Due to administration of chemotherapy in the inpatient setting, significantly longer hospital stays were observed in the CRT group compared to the RT group. Very short hospital stays in the RT group may, in turn, explain the observed decline of the VAS score at the end of therapy, as no supportive treatments requiring hospitalization could be administered in outpatients, and outpatient supportive care may not be adequately effective in elderly HNC patients. These findings suggest the benefits of generous inpatient treatment in order to maintain an adequate HRQoL and underline the importance of closely monitoring elderly HNC patients during treatment.

Taken together, our data show a gain of 0.76 QALYs by adding concurrent chemotherapy to RT in elderly HNC patients, given a careful selection of this vulnerable patient cohort.

Our analysis has some limitations pertaining to the rather small sample size and the single-center collection of HRQoL data. Given the exploratory character of our study, an adequate sample size calculation was not possible, and numbers of elderly HNC patients scheduled for concomitant CRT were limited even at a tertiary cancer center. Therefore, it cannot be ruled out that the sample size did not allow for detection of small differences in HI scores based on the EQ-5D-5L questionnaires [51]. Another limitation is the absence of a control group, which was unfortunately not ethically feasible for this analysis. Additionally, we cannot rule out a selection bias, as the patients included in this analysis may represent overly comorbid or low-performing patients, or patients of a more advanced age who were transferred to a tertiary cancer center due to risk factors. Our CE analysis is further limited by the fact that we could not model for a time horizon and account for costs of additional supportive interventions. As the analysis is based on German healthcare costs and the German billing system, there are limitations in transferring our findings to other countries and economic systems. Therefore, further inter-institutional research should expand these findings and corroborate our cost analyses of elderly HNC patients in the German healthcare setting.

In summary, our data provide evidence based on real-world data that curative CRT is cost effective compared to RT in selected elderly HNC patients in the German healthcare context. Further real-world ICERs are required from prospective studies with larger sample sizes in order to more precisely quantify the CE benefits.
